# Ongoing 5-year+ survival after multiple metastasectomies, followed by CAPOX plus bevacizumab, for metastatic colorectal cancer

**DOI:** 10.1186/s40792-020-00913-x

**Published:** 2020-06-26

**Authors:** Kozue Matsuishi, Yuji Miyamoto, Yukiharu Hiyoshi, Ryuma Tokunaga, Katsunori Imai, Hiromitsu Hayashi, Yoichi Yamashita, Naoya Yoshida, Hideo Baba

**Affiliations:** Department of Gastroenterological Surgery, Graduate School of Medical Sciences, Kumamoto University, 1-1-1 Honjo, Chuo-ku, Kumamoto, 860-8556 Japan

**Keywords:** Metastatic colorectal cancer, Metastasectomy, Chemotherapy, Long-term survival

## Abstract

**Background:**

Advancements in chemotherapy for metastatic colorectal cancer (mCRC) have improved long-term outcomes, and median survival currently exceeds 30 months. The recommended treatment for mCRC is multidisciplinary, including a combination of surgical resection and chemotherapy. In this study, we report the case of a patient who has survived for more than 5 years after an initial diagnosis of mCRC while undergoing first-line chemotherapy and six repeat metastasectomies.

**Case presentation:**

A 55-year-old man was diagnosed at our hospital with sigmoid colon cancer and hepatic metastasis. We performed laparoscopic sigmoidectomy and hepatic segmentectomy (segment 5 [S5] and S8). After resecting the primary tumor and liver metastasis, other metastases were found. Together with perioperative chemotherapy (CAPOX + bevacizumab), we performed repeated metastasectomies for liver metastasis (S4 and S7), pulmonary S1 metastasis, aortic lymph node metastasis, and right adrenal metastasis. With six metastasectomies, the patient has survived for more than 5.5 years.

**Conclusions:**

Multidisciplinary treatment extends survival and improves the quality of life in patients with mCRC. Planned surveillance after metastasectomy may also be necessary to promote the early detection of recurrence in these patients.

## Background

Colorectal cancer (CRC) is the third most commonly diagnosed cancer in the world and the third most common cause of cancer fatality [1]. Advances in chemotherapeutic treatment for metastatic colorectal cancer (mCRC) have improved long-term outcomes, and median survival currently exceeds 30 months. The recommended treatment for mCRC involves a multidisciplinary approach using combinations of surgical resection and chemotherapy. Recurrence after resection is common, but repeated resections are possible for some patients. In this study, we report a patient who has survived for more than 5 years after undergoing repeated metastasectomies and first-line chemotherapy.

## Case presentation

**Fig. 1:**
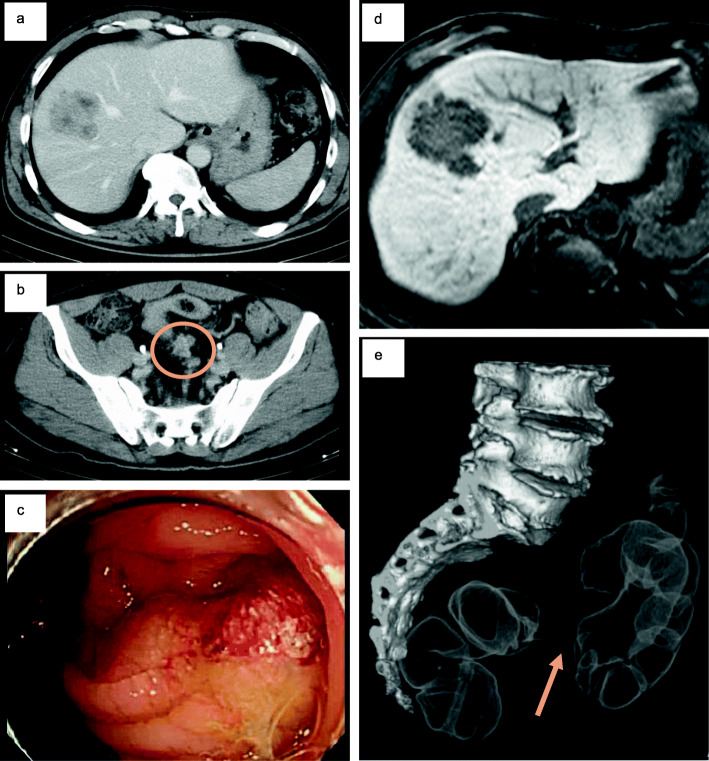
Pretreatment computed tomography (CT), magnetic resonance imaging (MRI), and colonoscopy. **a** Axial view of a liver metastasis on contrast-enhanced CT. **b** Axial view of regional lymph node metastases (circle) on CT. **c** Primary sigmoid colon cancer on colonoscopy. **d** Axial view of a liver metastasis on ethoxybenzyl diethylenetriamine-enhanced MRI. **e** Primary sigmoid colon cancer on CT-colonoscopy

**Fig. 2:**
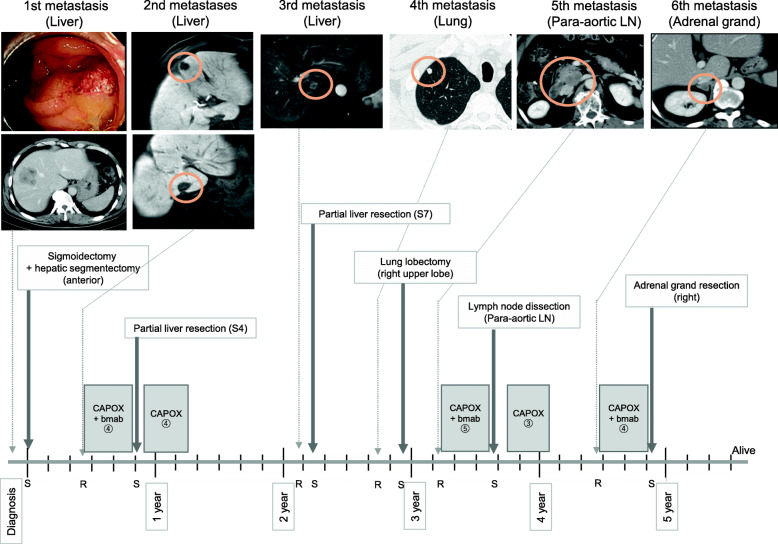
Clinical course of the patient’s metastases, surgeries, and other anticancer treatments. S, surgery; R, detection of recurrence

The patient was a 55-year-old man with no past medical history. In May 2014, a liver tumor was found via abdominal ultrasound screening, and he was referred to our department for further examination. As a metastatic liver tumor was suspected, he underwent colonoscopy, which located a circumferential type 2 lesion in his sigmoid colon. Biopsy revealed that the lesion was a well-differentiated type tubular adenocarcinoma (Fig. 1). Contrast-enhanced computed tomography (CT) revealed sigmoid colon cancer with regional lymph node metastases and a ring-shaped tumor measuring 50 × 48 mm^2^ in segment 8 (S8). Blood tests revealed no abnormalities in blood counts or biochemistry, but elevated CEA levels (9.3 ng/ml, 7.2 U/ml) were detected. First, we performed laparoscopic sigmoidectomy and hepatic segmentectomy (S5 and S8) in July 2014 for sigmoid colon cancer with liver metastasis. The pathological diagnosis illustrated that the primary and liver tumors were both well-differentiated adenocarcinoma (pT4aN1M1a [H2], pStage IV). These tumors were wild-type for both RAS and BRAF. Six months after the first surgery, multiple liver metastases (S3, 5 mm; S4, 17 mm; S6, 4 mm; S7, 9 mm) were found. Our multidisciplinary team decided to administer systemic chemotherapy following liver resection. After the patient received four courses of oxaliplatin and capecitabine plus bevacizumab (CAPOX + Bmab) as first-line chemotherapy for 3 months, CT revealed that the liver tumors in S3, S6, and S7 had disappeared, and the tumor in S4 had shrunk to 6 mm (response rate = 35%, partial response [PR]). We then resected the S4 tumor. After resection, the patient received four courses of CAPEOX as postoperative adjuvant therapy. The main adverse effects were peripheral neuropathy (grade 2) and hiccups (grade 1). Liver metastasis (S7, 14 mm) was detected 24 months after the first surgery. The multidisciplinary team decided not to administer preoperative chemotherapy because the tumor was small. Partial liver resection (S7) was performed. A fourth metastasis was detected in a right pulmonary nodule (S1) 32 months after the first resection, and it increased to 8 mm in size after 3 months. We determined that it was a lung metastasis and performed right upper lobectomy without preoperative chemotherapy because of its small size. Para-aortic lymph node metastasis was found 39 months after the first resection. Because the tumor was in contact with the duodenum and right renal vein, five courses of CAPOX + Bmab were administered. The aortic lymph node shrank from 24 to 11 mm in size (PR). Four months later, we decided that the lesion could be safely resected, and thus, aortic lymphadenectomy was performed. Three courses of chemotherapy were administered postoperatively. Right adrenal metastasis was found 53 months after the initial resection. Four courses of CAPOX + Bmab were administered, and the tumor slightly shrunk from 21 to 20 mm in size. Because treatment did not further reduce the tumor, we performed right adrenalectomy. While repeating CAPOX + Bmab chemotherapy, our multidisciplinary team resected the metastases. No features of sinusoidal syndrome were observed on clinical, pathological, or imaging examination after CAPOX + Bmab. This patient has survived for more than 5.5 years at the time of writing after undergoing six metastatic resections (Fig. 2).

## Discussion

To our knowledge, this is the first report of a patient with mCRC who survived for more than 5 years after undergoing multiple tumor resections and first-line chemotherapy, consisting of CAPOX plus Bmab in this case and six metastasectomies. We also believe this is the “first case” in which a patient continued CAPOX + Bmab as the first-line treatment while undergoing multiple resections. At that time, there was no concept of recommending a molecular target drug by the right and left colon, so we did not select an anti-EGFR antibody inhibitor. The choice of CAPOX + Bmab was tailored to the patient’s lifestyle. In addition, the patient selected the regimen. However, several reports have described patients with mCRC who achieved long-term survival after the resection of distant metastases [2–4]. Ottone et al. reported long-term survival in a patient after oxaliplatin-based chemotherapy and repeated thermoablation of liver metastases from CRC [5].

A multidisciplinary approach is optimal for long-term survival in patients with mCRC [6]. Although several novel chemotherapeutic and biological agents have been developed, resection remains the best option for achieving long-term survival for select patients with liver or lung metastases. However, most metastases are found after resection. The median time to recurrence is reportedly 16–19 months after resection among patients who received preoperative chemotherapy for liver metastasis of CRC [7]. Repeated resection for metastases might also improve long-term survival in certain patients with mCRC, including our current case [8, 9]. Solitary para-aortic lymph node metastasis after colorectal cancer surgery is a relatively rare recurrence type that is associated with a poor prognosis, but some reports described good outcomes using a multimodality approach including resection [10]. The adrenal gland is a common site of hematogenous metastasis of malignant tumors, but clinically, adrenal metastasis is often associated with multiple sites of metastasis. However, long-term survival has been reported in isolated cases of adrenal metastasis [11]. We suggest that patients with solitary lymph node or adrenal metastasis may benefit from complete surgical resection. Regularly scheduled CT may facilitate the early detection of metastases.

Sinusoidal syndrome reportedly occurred in 19% of patients who received preoperative oxaliplatin-based chemotherapy [12]; however, the incidence of sinusoids purportedly diminishes with the addition of Bmab to chemotherapy [13]. In this case, oxaliplatin + Bmab was used, but no advanced sinusoidal dilation was observed. The inclusion of Bmab might help protect against hepatotoxicity induced by oxaliplatin-based chemotherapy, in addition to its antitumor effect. However, Bmab is an angiogenesis inhibitor. Its withdrawal at 6 weeks before surgery is considered desirable because treatment with this drug delays wound healing.

Resections should be performed as early as possible after the tumor has shrunk in response to chemotherapy because all shrinkage occurs within eight courses with no further reduction expected [14] and because repeated chemotherapy increases the rate of postoperative complications [15]. Our patient responded well to chemotherapy, and tumor shrinkage was apparent. Preoperative chemotherapy can facilitate safer resection and systemic antitumor effects.

Several randomized studies explored the benefit of adjuvant 5-fluorouracil- or oxaliplatin-based chemotherapy following the curative resection of hepatic metastases [16–18], but the overall survival benefit of this strategy remains controversial [19]. However, we believe that perioperative chemotherapy could eradicate residual microscopic disease and improve survival for select patients. In general, perioperative chemotherapy is usually administered to patients with five or more metastases, extrahepatic metastases, or a short disease-free interval (< 6 months). For too small metastases, chemotherapy is not recommended because it may disappear after chemotherapy. In our case, we administered perioperative chemotherapy during the second, fifth, and sixth surgeries according to these criteria.

## Conclusions

In this study, we reported the case of a patient with mCRC who has survived for more than 5 years while receiving first-line chemotherapy and six repeated metastasectomies. Patients with mCRC may benefit from multidisciplinary treatment. Planned surveillance after metastasectomy may be necessary for the early detection of recurrence.

## Data Availability

All data generated or analyzed during this study are included in this published article.

## Abbreviations

CAPOX + Bmab: Oxaliplatin and capecitabine plus bevacizumab
CT: Computed tomography
mCRC: Metastatic colorectal cancer
MRI: Magnetic resonance imaging
